# Effects of Motivation, Basic Psychological Needs, and Teaching Competence on Disruptive Behaviours in Secondary School Physical Education Students

**DOI:** 10.3390/ijerph16234828

**Published:** 2019-12-01

**Authors:** Antonio Granero-Gallegos, Pedro Jesús Ruiz-Montero, Antonio Baena-Extremera, Marina Martínez-Molina

**Affiliations:** 1 Faculty of Education, University of Almería, 04009 Almería, Spain; agranero@ual.es; 2 Health Research Centre, University of Almeria,04009 Almería, Spain; 3 Faculty of Education and Social Sciences, Melilla Campus, University of Granada, 52006 Granada, Spain; 4 Faculty of Education, University of Granada, 18071 Granada, Spain; abaenaextrem@ugr.es; 5 Faculty of Education, University of Almería, 04009 Almería, Spain; marinamartinezmolina@gmail.com

**Keywords:** misbehaviours, adolescent, teaching, secondary school, multilevel regression models

## Abstract

Currently, disruptive and aggressive behaviours of a physical and verbal nature are a reality among adolescent students and a concern in the educational context. Therefore, the main objective of this research was to analyse the effects of perceived teaching competence, motivation and basic psychological needs on disruptive behaviours in secondary school PE students. The sample was composed of 758 adolescent students from seven public secondary schools. The following instruments adapted to physical education were used: The Disruptive Behaviours Questionnaire, The Evaluation of Teaching Competencies Scale, The Sport Motivation Scale, and The Basic Psychological Needs Scale. Multilevel regression models with the MIXED procedure were performed for data analysis. The results show that misbehaviour is more likely among male students and that disruptive behaviours decrease when a teacher is perceived as competent. Students with greater self-determined motivation are more likely to exhibit fewer behaviours related to low engagement and irresponsibility while amotivation increases the different disruptive behaviours in the classroom. In conclusion, it is proposed that educators work in line with the students’ needs by responding to their interests and that this will increase self-determined motivation.

## 1. Introduction

Nowadays, disruptive behaviours, and even aggressive physical and verbal behaviours are a reality among adolescent students in the educational context [1,2,3]. These misbehaviours are related to school failure [3] and are considered as predictors of future violent behaviours both in the school context [4,5] and in the formation of dangerous social environments [6]. The importance of this problem in the classroom has meant that more and more research has been directed towards the indiscipline and disruptive behaviours of primary and secondary school students as an indicator of teaching success or failure [5,7,8,9]. Moreover, these types of behaviours cause stress and work loss amongst teachers [10,11]

According to the self-determination theory (SDT) [6], student behaviours during a class may vary depending on a number of factors, such as motivation or the satisfaction of basic psychological needs (BPN). It considers that the educational context strongly influences student motivation and the control of disruptive behaviours, and is the reason for the proliferation of studies in this regard [12,13]. The explanation for certain personal decisions made by schoolchildren may be based on the relationship between motivation and social, family or personal factors [14]. However, it is also important to understand the students’ perception of the teacher’s competence if one wants to explain “why” there are bad behaviours in the classroom, and to try to reduce them or make them disappear. As Duchesne and Larose [15] state, if students perceive appropriate academic competencies during the learning process, their motivation increases and this prevents potentially disruptive behaviours. Our study addresses the issue in the context of physical education (PE). This subject stands out as promoting a climate of fun, motivation and stress avoidance amongst students, fostering a positive relationship between student satisfaction, the PE curriculum, and the school system itself [8,16,17,18].

SDT is one of the most important theories studied in relation to educational motivation, and is widely considered in the context of PE [6]. One must bear in mind that the motivational profile of adolescents during sports practice can help to understand their behaviour patterns [19]. It is important to note that human motivation and the functioning of personality are related within a given social context [6] and that there are three established levels: The highest level being self-determined motivation or intrinsic motivation, the intermediate level being extrinsic motivation, and the lowest level of amotivation. With regard to the first, this is understood as the pleasure of participating in an activity by the mere fact of enjoying it, being interested in it, or the personal satisfaction of carrying it out [20]. Intrinsic motivation in PE students produces a greater interest to participate both during the sessions and in their free time [21,22]. Extrinsic motivation, on the other hand, is the activity of a person aimed at meeting an external demand or to get a reward [6]. Finally, amotivation signifies a lack of motivation in practicing an activity, associated with the absence of intent [23], feeling incompetent when performing an activity [24], and the rejection of unexpected results [14].

Continuing with the SDT [6] to explain student behaviour in terms of their BPNs, the theory indicates that these needs may be primarily those of autonomy, competence, and relating to others—all necessary for psychological health. These same authors emphasize that each of the needs indicated plays a prominent role in the optimal development and experiencing of well-being in daily life. The need for autonomy is understood as the effort that people make to determine their own behaviour and the origin of their own actions; the need for capability is understood as controlling the outcome and the eagerness to experience effectiveness; and the need for relating to others alludes to concern for others, making others feel that they have an authentic relationship with oneself so that one might feel accepted and able to fraternize with others [25,26]. The satisfaction of BPN is associated with positive consequences, such as an improvement in autonomous motivation [27]. However, BPNs can also be frustrated and unsatisfied due to restrictions imposed by people in the surrounding environment [28], and in education this is manifested through disruptive behaviours or misbehavior [15,29]. The satisfaction of these three BPNs enhances learning in the PE class when adequate motivation is given to students, fostering greater academic commitment during the learning process [28,30].

At the same time, the figure of the teacher is key in the teaching process and the students’ perception of their teacher’s competence can be important in explaining bad behaviours in the classroom. Therefore, addressing the perception that students have of teacher competence together with the motivation and satisfaction of their BPNs in explaining disruptive behaviours in the classroom is the main novelty and contribution of this research to the scientific literature.

It should be remembered that in secondary education, the teacher is not evaluated by her/his own students, which does not help to ensure the internal quality of the teaching-learning process—the same goes for PE. It is therefore important that the methodology and approach used by the teacher is increasingly studied, considering, among other things, different competencies [31,32]. As some authors point out (e.g., [33]), the ways in which the PE teaching staff handles their classes regarding time, or the teaching environment, are essential factors in dealing with disruptive behaviours. Some uncertainty still exists when it comes to knowing how teachers achieve the skills necessary to carry out their teaching work, about how they confront the challenges of today’s society with their students, and how they assess the students’ needs [10]. As Christie, Quiñones, and Fierro [34] noted, a considerable number of teachers have doubts when having to make assessments although they would like to gain sufficient knowledge and competence to do so well. All of these aspects, such as the lack of competencies and teacher’s experience of dealing with difficult situations, are relevant in highlighting the importance of the teacher’s persona when it comes to handling disruptive behaviours [33].

Taking into account all of the above, the main objective of this study is to analyse the effects of perceived teaching competence, motivation, and basic psychological needs on disruptive behaviours in secondary school students in the PE class.

## 2. Materials and Methods

### 2.1. Participants

The design of this cross-sectional study is observational and descriptive. A non-probabilistic and convenience sample selection process was employed, based on the students that we were able to assess. This research included secondary school PE students—a total of 758 students (45.8% male) participated from seven secondary schools in the Murcia region. The age range was between 13 and 18 years old (*M* = 15.22, *SD* = 1.27; *M_boys_* = 15.20, *SD_boys_* = 1.29; *M_girls_* = 15.18, *SD_girls_* = 1.26).

### 2.2. Measurements

*Disruptive Behaviours in Physical Education*. The Disruptive Behaviour in Physical Education Questionnaire (CCDEF) [8] was used, the Spanish version of the original *Physical Education Classroom Instrument* (PECI) [33]. This version consists of 17 items that measure disruptive behaviours in PE students over five subscales: *Aggressive* (AGR) (2 items), *low engagement or irresponsibility* (LEI) (4 items), *fails to follow directions* (FFD) (4 items), *distracts or disturbs others* (DDO) (4 items), and *low self-management* (PSM) (3 items). A five-point Likert scale ranging from 1 (*never*) to 5 (*always*) was used for the responses. The internal consistency indexes were: AGR, Cronbach’s alpha (α) = 0.58, composite reliability = 0.81, average variance extracted (AVE) = 0.54; LEI, α = 0.73, composite reliability = 0.84, AVE = 0.74; FFD, α = 0.77, composite reliability = 0.94, AVE = 0.65; DDO, α = 0.81, composite reliability = 0.92, AVE = 0.80; PSM, α = 0.84, composite reliability = 0.96, AVE = 0.92. Given the low Cronbach’s alpha index and that the AGR subscale only consists of two items, this factor was ignored in the analyses we performed. The goodness-of-fit indices for the CCDEF scale using the confirmatory factorial analysis (CFA) were chi-squared (χ^2^)/degrees of freedom (*df*) = 3.61; incremental fit index (IFI) = 0.96; Tucker-Lewis index (TLI) = 0.95; comparative fit index (CFI) = 0.96; root mean square error of approximation (RMSEA) = 0.06; and standardized root mean square residual (SRMR) = 0.06.

Teaching competence. The Spanish version of the *Evaluation of Teaching Competencies Scale adapted to Physical Education* (ETCS-PE) [31] was used, adapted from the original *Evaluation of Teaching Competencies Scale* [35]. This consists of eight items that measure the students’ perception of teacher effectiveness. A seven-point Likert scale ranging from low (1, 2), medium (3, 4, 5), and high (6, 7) was used for the responses. The internal consistency indices were: α = 0.86, composite reliability = 0.86, AVE = 0.59. The goodness-of-fit indexes of the scale using the CFA were: χ^2^/df = 1.80; IFI = 0.99; TLI = 0.99; CFI = 0.99; RMSEA = 0.03; SRMR = 0.02.

*Sport Motivation Scale* (SMS). The Spanish version of the original *Sport Motivation Scale* [36], adapted to PE [37] was used. This consists of 28 items that measure the different types of motivation established by the SDT [23], which suggests the multidimensional explanation of motivation: *Amotivation* (AMO), *extrinsic motivation* (EM) (12 items), and *intrinsic motivation* (IM) (12 items). Responses were collected on a Likert scale ranging from 1 (*totally disagree*) to 7 (*fully agree*). The internal consistency found in this study was: IM, α = 0.91, composite reliability = 0.99, AVE = 0.92; EM, α = 0.91; composite reliability = 0.99, AVE = 0.88; AMO, α = 0.75, composite reliability = 0.85 and AVE = 0.58. The goodness-of-fit indexes for the scale using AFC were: χ^2^/df = 3.02; IFI = 0.96; TLI = 0.97; CFI = 0.98; RMSEA = 0.05; SRMR = 0.04.

*Basic Psychological Needs*. The Spanish version of the *Basic Psychological Needs Measurement Scale* [25] was used, adapted from the original *Basic Psychological Needs in Exercise Scale (BPNES)* [38]. This consists of 12 items grouped into three dimensions of four items each: *Autonomy* (AUT), *competence* (COM), and *relatedness* (REL). A Likert scale ranging from 1 (*totally disagree*) to 5 (*totally agree*) was used for the responses. The internal consistency found in this study was: AUT, α = 0.82, composite reliability = 0.88, AVE = 0.65; COM, α = 0.78; composite reliability = 0.88, AVE = 0.64; REL, α = 0.75, composite reliability = 0.90, AVE = 0.69. The goodness-of-fit indexes for the scale using AFC were: χ^2^/df = 3.88; IFI = 0.96; TLI = 0.95; CFI = 0.95; RMSEA = 0.05; SRMR = 0.06.

### 2.3. Procedure

Permission to carry out the work was obtained from the competent bodies, whether at the secondary schools or at the university. The parents and the adolescents were informed of the protocol and the study’s subject matter. The informed consent of both was an indispensable requirement to participate in the research. The tools measuring the different variables were administered in the classroom by the researchers themselves, without the teacher present. All participants were informed of the study objective, the voluntary and confidential nature of the responses and the data management, as well as their rights as participants thereof, based on the document approved by the Ethics Committee of the University of Murcia (REF-45-20012016).

### 2.4. Statistical Analyses

The descriptive statistics of the items, the correlations, and the internal consistency of each subscale were calculated, as well as the asymmetry and kurtosis, the statistics of which achieved values of between −0.78 and 1.67. Since the sample was made up of different subgroups (e.g., school and course), which might constitute a breach of the independence of observations assumption, it was considered relevant to use regression and multilevel modelling analysis (MLM) [39], taking into account the variables of the participants’ individual characteristics (level 1) and the context variables (level 2) [40]. The grouping or level of the students’ school and course was considered as a random effect. Different multilevel regression models were tested according to the different combinations of school and course levels, including a zero model in each of the CCDEF subscales. To check the effects of teaching competence, motivation, and basic psychological needs on each of the factors of disruptive behaviours, which acted as dependent variables, different models were tested in which the sex and age variables were also taken into account.

The multilevel regression analyses were performed with the SPSS 22.0 MIXED (IBM, Chicago, IL, USA) procedure using the restricted maximum likelihood estimation method. The –2 log-likelihood (–2LL) and the Akaike information criterion (AIC) [41,42] statistical equations were used as goodness-of-fit measures between models. In addition, to indicate the absence of lost values and the assumption of normality of the residuals for all the models, these were checked in all cases. The reduction or exclusion of possible interactions between the model-independent variables was carried out using the goodness-of-fit measures mentioned above, along with checking the hypothesis contrasts associated with the intersection parameters.

## 3. Results

### 3.1. Descriptive and Correlation Analysis

As shown in Table 1, the average scores of the different scales achieved moderate values, although these were quite low among the factors for disruptive behaviours. In terms of motivation, the *intrinsic motivation* score was higher and the *amotivation* score was lower. Among the basic psychological needs, *competence* presented higher mean values while the *autonomy* values were lower. Regarding disruptive behaviours, *low engagement or irresponsibility* presented the highest means while *poor self-management* was the lowest.

In Table 1, one can observe how age did not correspond significantly, from the statistical standpoint, to any subscale. The positive and statistically significant correlations between *teaching competence* and *intrinsic motivation*, *extrinsic motivation* and *autonomy* stand out, as do those between *intrinsic motivation* and *extrinsic motivation*, and between the four CCDEF subscales. In contrast, disruptive behaviours presented negative correlations with the other dimensions, except for *motivation*, highlighting the statistically significant relationships with *teaching competence*, *intrinsic motivation*, and *relatedness*. Conversely, for *amotivation*, the correlations of the CCDEF subscales (LEI, FFD, DDO, PSM) were positive and statistically significant.

### 3.2. Multilevel Regression Analysis

A multilevel regression model without predictor variables (the null model, M0) was tested. M0 served to evaluate the predictive gain of models in which the predictor variables were included, both the constant effect and the random effects of school and course. Based on the goodness-of-fit values of the evaluated models, we found that the model including the school and the constant (M1) had the greatest effect on the independence of the observations: M0, −2LL = 1990.22; M1, −2LL = 1964.35 (χ^2^ = 25.87; df = 1; *p* < 0.0001). An intraclass correlation coefficient (ICC) was calculated: ICC = 0.12 in LEI; ICC = 0.08 in FFD; ICC = 0.17 in DDO; and ICC = 0.07 in PSM. This indicates that between 7% and 17% of the total variability, depending on the dependent variable, is attributable to the difference between the mean of the different schools.

Based on the M1 results, different models were tested considering the grouping or level of the school. In these models, the dependent variable (the four CCDEF factors) was changed and, in each of them, the independent level 1 variables (model 2, M2) were introduced into the regression equation, namely sex, age, ETCS-PE, IM, EM, AMO, AUT, COM, and REL.

Table 2 shows the results of the models in which the dependent variable was *low engagement or irresponsibility*. In M2, one can observe that the effects of age were not statistically significant, nor were the EE, AUT, and REL effects. In contrast, the effects of sex, ETCS-PE, IM, AMO, and COM were statistically significant. Next, model 3 (M3) was estimated, from which the variables whose effects were not statistically significant on *low engagement or irresponsibility* were removed. The goodness-of-fit indexes indicated a better fit for M3 than for M2 (χ^2^ = 12.27; df = 5; *p* < 0.05). In M3, the effect of the student’s own competence (COM) was not statistically significant so it was eliminated to estimate model 4 (M4) in which the variables that did have predictive and statistically significant effects were included. M4 did not represent a statistically significant improvement over M3 (χ^2^ = 1.89; df = 4; *p* > 0.05), so M3 was adapted as the most suitable model. Thus, it was proven that, for the boys, it is more likely that irresponsibility will occur as disruptive behaviour. In addition, as greater teacher competence is perceived, the student has greater intrinsic motivation, which means irresponsibility is more likely to decrease. However, if amotivation increases, irresponsibility and poor self-management is more likely to increase.

The results of the models in which the dependent variable was *Fails to follow directions* are shown in Table 3. In M5, the effects of age, MI, ME, and COM were evaluated and they did not prove to be statistically significant. Therefore, these variables were removed from M6, which included sex, ETCS-EF, AMO, AUT, and REL. The goodness-of-fit indexes indicated a worse fit for M6 than for M5 (χ^2^ = 1.62; df = 5; *p* > 0.05). In this way, the effect of sex on *disobeying the rules* was also determined, and it was significantly more likely to occur amongst boys than amongst girls. Furthermore, disobedient behaviour is more likely to decrease as more teacher competence is perceived and social relations improve with the others. In contrast, increased amotivation would mean greater disobedience is more likely amongst the boys, as has been indicated. Feeling more autonomous can also lead to increased disobedience, so every effort must be made to positively channel that autonomy.

The same was process was carried out for the *Distracts or disturbs others* variable and the results of the models are shown in Table 4. After the variables were removed whose effects were not statistically significant on M7 (age, MI, ME, and COM), M8 did not show a significant improvement (χ^2^ = 7.73; df = 5; *p* > 0.05), so the M7 results were accepted. The sex variable continued to present statistically significant values with boys more likely to be *disruptive in the class environment*; however, in this case too, greater teacher competence and improved social relations with classmates would be predictors of less disruption in the classroom. As with disobeying the rules, amotivation and feeling more autonomous can lead to an increase in disruptive behaviour in the classroom.

The results for low personal self-control are shown in Table 5. M9 was estimated and the variables whose effects were not statistically significant (age, intrinsic motivation, extrinsic motivation, competence, and relatedness) were removed to estimate M10. However, this model did not present a better fit than the previous model (χ^2^ = 0.37; df = 4; *p* > 0.05) so the M9 results were accepted. The predictive values are even higher in the case of low personal self-control amongst males (0.35) and these are accentuated if there is amotivation and a feeling of more autonomy. Only increased teaching competence is shown as a statistically significant predictor for greater personal self-control on the part of students.

## 4. Discussion

The objective of this work has been to analyse the effects of teaching competence, motivation, and basic psychological needs on the disruptive behaviours of secondary school PE students. Accordingly, we have tried to provide answers to some of the main problems that exist today in classrooms such as behavioural and discipline issues [43], which are common across the different curriculum subjects [44,45]. The present study shows that the various disruptive behaviours are more likely to occur in males. In addition, it is interesting to note that when a teacher is more competent, there are less likely to be behaviour problems amongst students. Regarding motivation, it has been found that, among students with more self-determined motivation, fewer low engagement and irresponsibility behaviours will occur whereas amotivation will increase the various disruptive behaviours in the classroom. On the other hand, it should be pointed out that, amongst the most autonomous students, disciplinary problems are more likely. Therefore, we will now analyse what characteristics should accompany student autonomy during the learning process.

For the students in the present study, the boys were always the ones presenting the greatest disruptive behaviours in the PE class. The results are in line with previous studies where boys showed behaviours more associated with irresponsibility and low commitment in the PE class than girls did [46,47]. Conflict situations in class are often associated with boys because of the importance they place on peer differences, competitiveness, continually striving to win or discrimination of a motor skill [48]. The authoritarian nature that boys can sometimes exhibit, coming from the desire to exaggerate their masculinity in PE classes [49], can lead to disobeying the rules laid down by the teachers. All this contrasts with the role of girls and their behaviour in PE classes, where they show more passive behaviours and a respect for or commitment to the class group and to the teachers [21,50].

On the other hand, PE classes at any educational level are often related to dominant situations and male hegemony, which increases gender disparity [51,52]; this can lead to situations of inequality or taunts amongst students due to low personal self-control. This study illustrates precisely that boys have less personal self-control than girls, coinciding with a previous study of Spanish teenagers where male self-control was difficult to achieve and therefore led to a disruptive environment in the class [46]. Exploratory studies trying to understand power and gender, especially in boys, explain the importance they attach to appearance and how to act in front of others [49,53]. Therefore, this could explain the relationship between the male gender and a disruptive class atmosphere that we observed in our study.

The various disruptive behaviours are present on a daily basis in schools and are of great concern to the different educational agents, such as teachers [3,43]. This study highlights the importance of students perceiving their teachers as competent and prepared to deal with everyday life, as this implies that there is less likelihood of behavioural problems related to classroom instruction; the same conclusion has been reached in various studies [54]. These behaviours affect order and disrupt both the functioning and the environment within the class, which has consequences on the students themselves and on the learning context [3], some of which have been studied in this research—low engagement or irresponsibility, failing to follow directions, distracts, or disturbs others and poor self-management. Although these behaviours are highlighted from the teacher’s perspective in current studies [3,55], this paper also highlights the importance of the teacher’s own competence in terms of good communication, work awareness, creativity, feed-back, individual consideration, professionalism, problem solving, and social awareness. These aspects have already been the focus of various works [16,35] in linking improved student self-control with the students’ perception of adequate teaching competence. This study contributes to the scientific field by stressing the importance of the figure of the competent teacher in preventing or controlling bad behaviour in the classroom. Therefore, this should influence all aspects of teacher training, including the initial training, to ensure that they become as professionally competent as possible.

The results obtained in this study show how low commitment and irresponsibility on the part of the students relates to the lack of teaching competence on the part of PE teachers. It is therefore necessary for PE teachers to offer their students varied and motivating tasks and activities which add quality to the learning and encourage their interest [21,46]. Moreover, students would show openly their motivation and would increase their personal abilities with respect to contents of PE lessons and therefore, there will be an decrease of disruptive behaviours [3].The PE curriculum is one of the most effective of all the school curricula at promoting greater student concentration and commitment but only provided that the appropriate methodological strategies are used by the teachers [56].

With regard to behaviours centered on disobeying the rules, discipline problems in the educational field are common in all areas and subjects that make up the school curriculum [45]. This has serious implications for the teaching-learning process because, among other reasons, they limit the time dedicated to learning [7]. Consequently, PE teachers must create an appropriate classroom environment by fluidly communicating with their students and being as effective as possible when faced with any problematic situation that arises [35,57] using verbal and highly rational strategies, as recommended by some authors [4].

Many teachers and students are concerned about the disorder and the risk of a poor environment in classrooms and schools. The entire educational community is alerted to the high incidence of behavioural problems occurring in schools—drug use, cheating, insubordination, absenteeism, intimidation, etc.—all of which have serious repercussions on the educational community. Behavioural problems in the classroom have long been one of the most discussed and analysed topics in the educational field [58,59,60,61].

The degree to which students can disrupt an adequate classroom environment is related to their positive perception of teacher competence, as can be seen in the present study. Traditional and inflexible methodologies on the part of the teacher, justifying higher motor performance and filling the time with physical work, are a cause of dissatisfaction and disruption of the PE class environment [62,63]. An understanding and empathetic attitude by teachers, promoting the inclusion of all students in the PE class through the sharing of responsibilities and continuous dialogue are key to better bonding among the students in the class and between the class and the teachers themselves [63,64,65].

Likewise, greater personal self-control on the part of the students is related to higher levels of teaching competence. Various symptoms related to the students’ lack of control are linked to dissatisfaction with the school, which can even lead to them dropping out of school [66]. Our results are in line with those obtained in a study measuring disruptive behaviours in Spanish adolescent students aged 12–18 in the PE area [8], which concluded that teachers’ skills when managing different behaviours and pre-planning are fundamental elements in strengthening the students’ personal self-control and controlling disruptive behaviours.

The importance of motivation in the PE class determines the students’ satisfaction with this subject [16]. In our study, greater intrinsic motivation on the part of the students was accompanied by less irresponsible behaviours. Several studies have provided similar results to ours with respect to intrinsic motivation [8,36,67,68], favouring in turn a focus and concentration in the PE class and fewer disruptive behaviours [69]. In addition, if one works to promote student responsibility and empowerment through greater autonomy and trust on the part of teachers, serious misbehaviours could decrease considerably, fostering an optimal classroom and learning environment [21,68,70].

On the other hand, amotivation or lack of motivation in the students is an essential criterion when it comes to understanding the level of satisfaction felt by the PE students [71]. If amotivation is high, there tends to be a decrease in engagement and an increase in irresponsible behaviours in the PE class, where no minimal effort exists to commit to the tasks proposed by the teachers [6,71]. A lack of competence when performing physical activities, or not valuing the activities proposed in the PE class are other examples of amotivation [6]. If amotivation can be controlled or minimized by the PE teachers and, at the same time, intrinsic motivation is properly fostered, student commitment can lead to the desired learning of the contents proposed in the class [72,73]. In our study, disobeying the rules, especially for boys, is related to amotivation, explained by the frustration and fear that they may experience when performing a physical test [74,75] or by their rejection of the PE teachers for various reasons [74], many of which we have already commented on. To all that is cited above, one should add that the lack of information when performing physical activities or overcoming a physical test in the PE class can involve amotivation by the students and a respective disobeying of the rules. Therefore, it is necessary to inform the students about the class objectives and to describe the necessary criteria for carrying out any physical activity [16,71].

The basic needs of the students should be met as the PE class develops. Otherwise, students may see no reason to follow the normal development of the class and begin to disrupt the classroom environment [76]. If continued student participation and perseverance in the work are satisfactorily reciprocated by the PE teacher, an optimal response is established which contributes to increased student motivation [77]. Sometimes, the problem is simply that we do not pick up on the signs that our students give off during the course of the PE classes. Therefore, it is necessary to understand the students’ perspectives regarding the PE class and towards the teachers themselves in order to interpret the low level of control they feel, and the way they act towards their classmates and the teacher [33].

It is also necessary to adequately manage the autonomy of students or intrinsic motivation in the PE class given that, in this study, it is related to most of the disruptive behaviours. Intrinsic motivation presents in students who act only according to their own interests and values, who feel the need to make their own decisions in the different activities and tasks proposed [77,78]. The intrinsic motivation of the students should be positively channeled to meet their needs and to enhance them in a way that arouses their interest and curiosity for achieving a desired goal [79]. In this way, disobeying the class rules could be diminished.

A great deal of responsibility lies with the teachers themselves. They should adopt more flexible attitudes towards their students and be more open to understanding their feelings and desires [80], giving them more opportunities to perform tasks [81], using more positive language and inviting them to perform tasks [29], and fostering dependent relationships between themselves and the students [71]. Moreover, a higher teacher competence must contain qualities such as availability, communication, conscientiousness, creativity, feedback, individual consideration, professionalism, problem-solving, and social awareness [8,33].

The role that teaching competence plays in the students’ disruptive behaviours is also discussed in this study and, of course, this can also influence the intrinsic motivation.

It should be noted that fun is linked to the perception of intrinsic motivation [82], and that this can get the students to obey the rules more and refrain from continually disrupting the PE class. The students in our study presented a high level of intrinsic motivation, which is linked to greater disobedience, one of the causes of which might be precisely the lack of fun or feeling of well-being during the classes. In a study conducted on Finnish teenage students, their motivation was linked to fun and their regular adherence to class rules [78]. In another study on a similar sample [79], the boys who felt their self-motivated needs were satisfied and the girls who felt that their relationship needs were met, showed fewer negative experiences and greater autonomy. If there is no fun or satisfaction in the PE class, students may have difficulty controlling their impulses in the classroom environment or towards the teacher. If teachers provide adequate feedback to help modify and improve the proposed task, this will also help to control the perception of the students. There will also be the beneficial response of enhancing positive motivation and satisfaction for teachers and in the classroom environment [78,83]. Therefore, supporting of teacher to student’s autonomy is necessary to improve the motivation of students during the PE lessons [12,27,37].

Amotivation or the explanation of an intrinsic motivation in a given situation might be caused by problems in relating to others, leading to a lack of control in a group situation or when interacting with another classmate. The frustration of basic needs when interacting with others, or in the normal development of a group environment, causes rigid patterns of behaviour that affect interactions with others [15,29]. This can lead to continuously stressful situations for the students and, therefore, cause them to avoid achieving a goal proposed by the teacher to protect themselves against any threat to their psychological integrity [15,84]. Therefore, another possible consequence of the relationship between low personal control and interacting with one’s classmates could be the stressful situations experienced during PE classes. Finally, a binary model of students and teachers where the interaction between them is closer could minimize possible disruptive behaviours of students because of the relationship created in the classroom´s daily life [85].

## 5. Conclusions

The results obtained in this study show how disruptive behaviours in secondary school PE students can be produced by the type of motivation they develop, the satisfaction of their basic psychological needs, and by how they perceive teaching competence. Furthermore, our results support the vast majority of previous studies conducted on Spanish adolescent students looking at disruptive behaviours and the effects of motivation, basic needs, and teaching competence. It should be noted that, among all the independent variables addressed in this study, the sex of the students, the perceived teaching competence, and amotivation have been present in all the relationships established with the four disruptive behaviours under study (the dependent variables being: Irresponsibility and low commitment, disobeying the rules, disrupting the class environment, and low personal self-control).

Consequently, future research on samples with similar characteristics to those evaluated in the present study should focus the teaching-learning process on increasing the motivation of PE students. Amotivation is one of the main causes of disruptive behaviours in the class along with the students’ perception of teacher competence, which is also important. In addition, boys are more likely to exhibit inappropriate behaviours than girls; this agrees with most national and international studies on similar samples and those looking at PE classes. Therefore, this aspect should be considered by the teacher when dealing with classroom management. It should be noted that this study may be relevant for teachers who intend to take PE sessions with secondary school students.

In summary, the specific training that teachers are given addressing bad behaviours in the classroom must begin in the initial training (university teacher training courses) and continue throughout their professional career in order to respond to the motivational demands and the basic psychological needs of PE students. We also propose working in line with students’ needs to respond to their interests and to increase their self-determined motivation. In this regard, it is also important to address the teaching process with current pedagogical models that relate to active methodologies. Finally, we also propose strengthening both the coordinated work between teachers and the tutoring employed when addressing these bad behaviours.

## Figures and Tables

**Table 1:** Descriptive statistical and correlations.

Subscales	1	2	3	4	5	6	7	8	9	10	11	12
1. Age	-	0.05	0.02	0.01	−0.06	−0.02	−0.00	−0.01	0.02	0.04	0.00	0.05
2. ETCS-EF		-	0.43 **	0.39 **	0.06	0.41 **	0.25 **	0.19 **	−0.21 **	−0.15 **	−0.12 **	−0.10 **
3. IM			-	0.82 **	0.17 **	0.61 **	0.56 **	0.45 **	−0.25 **	−0.19 **	−0.10 **	−0.08 *
4. EM				-	0.29 **	0.58**	0.53 **	0.43 **	−0.18 **	−0.19 **	−0.05	−0.04
5. AMO					-	0.15**	−0.03	−0.03	0.18 **	0.26 **	0.24 **	0.20 **
6. AUT						-	0.67 **	0.50 **	−0.14 **	−0.07	−0.01	0.04
7. COM							-	0.57 **	−0.20 **	−0.16 **	−0.08 *	−0.04
8. REL								-	−0.15 **	−0.19 **	−0.14 **	−0.09 *
9. LEI									-	0.70 **	0.64 **	0.59 **
10. FFD										-	0.71 **	0.66 **
11. DDO											-	0.79 **
12. PSM												-
*M*	15.20	5.36	4.94	4.85	3.72	3.18	3.56	3.83	2.00	1.65	1.49	1.42
*SD*	1.36	1.16	1.36	1.25	1.57	0.94	0.89	0.90	0.90	0.84	0.79	0.83

Note. *M* = mean; *SD* = standard deviation; ETCS-PE = teaching competence; IM = intrinsic motivation; EM = extrinsic motivation; AMO = amotivation; AUT = autonomy; COM = competence; REL = relatedness; LEI = low engagement or irresponsibility; FFD = fails to follow directions; DDO = distracts or disturbs others; PSM = poor self-management; ** *p* < 0.01; * *p* < 0.05.

**Table 2:** Regression and multilevel modelling analysis: Estimations and adjustments. Dependent variable: Low engagement or irresponsibility.

	Models								
	M2			M3			M4		
	Estimation (Error)	t	(sig.)	Estimation (Error)	t	(sig.)	Estimation (Error)	t	(sig.)
Variables									
Sex	0.26 (0.06)	6.76	0.000	0.27 (0.06)	4.40	0.000	0.25 (0.06)	4.14	0.000
Age	0.03 (0.02)	1.35	0.178						
ETCS-PE	−0.09 (0.03)	−3.15	0.002	−0.08 (0.03)	−2.78	0.006	−0.08 (0.03)	−2.84	0.005
IM	−0.17 (0.05)	−3.56	0.000	−0.14 (0.03)	−4.75	0.000	−0.17 (0.03)	−6.70	0.000
EM	0.02 (0.05)	0.47	0.642						
AMO	0.11 (0.02)	5.31	0.000	0.12 (0.02)	5.98	0.000	0.13 (0.02)	6.39	0.000
AUT	0.08 (0.05)	1.62	0.105						
COM	−0.12 (0.05)	−2.37	0.018	−0.08 (0.04)	−1.86	0.063			
REL	0.00 (0.04)	0.10	0.920						
Adjustment Statistics									
Deviance (–2LL)	1902.95			1890.68			1889.65		
AIC	1904.95			1892.68			1891.65		

Note. Sex: 0 = female; 1 = male; M2 = model 2; M3 = model 3; M4 = model 4; ETCS-PE = teaching competence; IM = intrinsic motivation; EM = extrinsic motivation; AMO = amotivation; AUT = autonomy; COM = competence; REL = relatedness; –2LL = –2log-likelihood; AIC = Aikake information criterion.

**Table 3:** Regression and multilevel modelling analysis: Estimations and adjustments. Dependent variable: Fails to follow directions.

	Models					
	M5			M6		
	Estimation (Error)	t	(sig.)	Estimation (Error)	t	(sig.)
Variables						
Sex	0.25 (0.06)	4.48	0.000	0.23 (0.06)	4.00	0.000
Age	0.04 (0.02)	1.95	0.052			
ETCS-PE	−0.07 (0.03)	−2.46	0.014	−0.10 (0.03)	−3.60	0.000
IM	−0.08 (0.04)	−1.82	0.069			
EM	−0.06 (0.05)	−1.25	0.214			
AMO	0.15 (0.02)	7.51	0.000	0.13 (0.02)	7.13	0.000
AUT	0.12 (0.05)	2.67	0.008	0.02 (0.04)	0.41	0.681
COM	−0.06 (0.05)	−1.29	0.197			
REL	−0.10 (0.04)	−2.48	0.013	−0.16 (0.04)	−4.38	0.000
Adjustment Statistics						
Deviance (–2LL)	1781.77			1780.15		
AIC	1783.77			1782.15		

Note. Sex: 0 = female; 1 = male; M5 = model 5; M6 = model 6; ETCS-PE = teaching competence; IM = intrinsic motivation; EM = extrinsic motivation; AMO = amotivation; AUT = autonomy; COM = competence; REL = relatedness; –2LL = –2log-likelihood; AIC = Aikake information criterion.

**Table 4:** Regression and multilevel modelling analysis: Estimations and adjustments. Dependent variable: Distracts or disturbs others.

	Models					
	M7			M8		
	Estimation (Error)	t	(sig.)	Estimation (Error)	t	(sig.)
Variables						
Sex	0.25 (0.06)	4.39	0.000	0.24 (0.06)	4.31	0.000
Age	0.01 (0.02)	0.64	0.522			
ETCS-PE	−0.06 (0.03)	−2.28	0.023	−0.08 (0.03)	−3.06	0.002
IM	−0.07 (0.04)	−1.52	0.130			
EM	−0.01 (0.05)	−0.24	0.810			
AMO	0.12 (0.02)	6.17	0.000	0.10 (0.02)	6.23	0.000
AUT	0.10 (0.04)	2.37	0.018	0.05 (0.04)	1.41	0.159
COM	−0.02 (0.05)	−0.39	0.695			
REL	−0.10 (0.04)	−2.73	0.006	−0.13 (0.03)	−3.87	0.000
Adjustment Statistics						
Deviance (–2LL)	1736.56			1728.83		
AIC	1738.56			1730.83		

Note. Sex: 0 = female; 1 = male; M7 = model 7; M8 = model 8; ETCS-PE = teaching competence; IM = intrinsic motivation; EM = extrinsic motivation; AMO = amotivation; AUT = autonomy; COM = competence; REL = relatedness; –2LL = –2log-likelihood; AIC = Aikake information criterion.

**Table 5:** Regression and multilevel modelling analysis: Estimations and adjustments. Dependent variable: Poor self-management.

	Models					
	M9			M10		
	Estimation (Error)	t	(sig.)	Estimation (Error)	t	(sig.)
Variables						
Sex	0.35 (0.06)	5.95	0.000	0.34 (0.06)	5.76	0.000
Age	0.04 (0.02)	−2.05	0.073			
ETCS-EF	−0.06 (0.03)	−2.05	0.041	−0.08 (0.03)	−2.83	0.005
IM	−0.07 (0.04)	−1.45	0.146			
EM	−0.04 (0.05)	−0.73	0.468			
AMO	0.10 (0.02)	4.98	0.000	0.09 (0.02)	5.08	0.000
AUT	0.16 (0.05)	3.52	0.000	0.03 (0.03)	0.90	0.367
COM	−0.03 (0.05)	−0.66	0.509			
REL	−0.07 (0.04)	−1.88	0.060			
Adjustment Statistics						
Deviance (–2LL)	1811.04			1810.67		
AIC	1813.04			1812.67		

Note. Sex: 0 = female; 1 = male; M9 = model 9; M10 = model 10; ETCS-PE = teaching competence; IM = intrinsic motivation; EM = extrinsic motivation; AMO = amotivation; AUT = autonomy; COM = competence; REL = relatedness; –2LL = –2log-likelihood; AIC = Aikake information criterion.
